# An assessment of the likely acceptability of vaginal microbicides for HIV prevention among women in rural Ghana

**DOI:** 10.1186/1472-6874-12-40

**Published:** 2012-11-01

**Authors:** Martha A Abdulai, Frank Baiden, George Adjei, Samuel Afari-Asiedu, Kwame Adjei, Charlotte Tawiah, Sam Newton

**Affiliations:** 1 Kintampo Health Research Centre, P. O. Box 200, Kintampo, Ghana

**Keywords:** Women, Microbicide, HIV, AIDS, Africa

## Abstract

**Background:**

The findings of the CAPRISA tenofovir studies have raised expectations that soon an approved microbicide would be available. However it is in only a limited number of countries in sub-Saharan Africa that the acceptability of microbicides has been evaluated. We conducted a study to assess the acceptability of vaginal microbicides among women in rural Ghana.

**Methods:**

The study employs a mixed method design, using cross-sectional survey and focus group discussions to further understand issues related to awareness and attitudes towards microbicide development, acceptability and perceived partner attitudes among pregnant women attending antenatal clinic in two health facilities in the Kintampo North municipality of Ghana. We used logistic regression to identify possible predictors of microbicide acceptability among the women surveyed.

**Results:**

Although only 2% of the 504 women were aware of the development of microbicides, 95% were willing to use one when it became available. The cost of a microbicide that will be considered affordable to 50% of women was US$0.75. Although there were concerns about possible wetting effect, gel or creams were the most preferred (68% of women) formulation. Although 71% thought their partners will find microbicide acceptable, apprehensions about the feasibility of and consequences of failed discreet use were evident. 49% of women were concerned about possible negative effect of microbicide on sexual pleasure. Perceived partner acceptability (O.R. =17.7; 95%C.I. 5.03-62.5) and possibility of discreet use (O.R. =8.9 95%C.I. 2.63-30.13) were the important predictors of microbicide acceptability.

**Conclusion:**

Achieving microbicide acceptability among male partners should be made a part of the promotive interventions for ensuring effective use among women in rural Ghana.

## Background

According to the 2010 UNAIDS report on the global AIDS epidemic, 33.3 million people live with HIV and 1.8 million deaths due to AIDS occurred in 2009. The total number of new infections in 2009 was 2.6 million. Sub-Saharan Africa remains the hardest hit by the HIV/AIDS pandemic and access to HIV prevention, treatment, care and support in this part of the world remains a major priority in global health [1,2].

Heterosexual transmission accounts for more than 80% of all new HIV infections in sub-Saharan Africa [3]. Traditional HIV preventive methods such as condom use, abstinence and “being faithful” are not always feasible due to various socio-economic and cultural factors [4-6]. Power inequity in relationships and intimate partner violence also increase the risk of HIV infection in young women [7]. Because of the male-dominated character of societies in most of sub-Saharan Africa, new and innovative approaches that empower women in HIV prevention are needed [8]. Such approaches should however be carefully evaluated for their acceptability and potential for seamless incorporation into national HIV programs [9].

Microbicides are compounds that can be applied locally to genital mucosal surfaces to protect against sexually transmitted infections (STIs) including HIV [10]. They can be formulated as gels, creams, films, or suppositories, and may or may not have spermicidal activity (contraceptive effect). Potential microbicides that have been investigated to date have been formulated to act through various mechanisms. This includes acting as physical barriers to keep HIV and other pathogens from reaching target cells, maintaining an acidic pH in the vagina, enhancing the natural defence mechanisms of the vaginal mucosa and stripping pathogens of their outer covering [11,12]. As at the time of writing, the major focus of HIV research was evaluating how antiretroviral agents may be used as microbicide. The candidate microbicide that is in the most advanced stage of development is the tenofovir gel [13,14].

After many years of research, characterised by repeated unfavourable outcomes, the announcement of the findings of the CAPRISA (Centre for the AIDS Program of Research in South Africa) study in July 2010 represented a major breakthrough [15]. The study tested the safety and effectiveness of 1% tenofovir gel among nearly 900 women at two sites in South Africa. It found that Tenofovir gel reduced HIV infection by 39%. The VOICE (Vaginal and Oral Interventions to Control the Epidemic) trial which was anticipated to strengthen the evidence from the CAPRISA study, was however discontinued by its Data Safety Monitoring Board (DSMB). Even though it found the tenofovir gel safe to use, it did not find it effective in preventing HIV infection among women in South Africa, Zimbabwe and Uganda [16]. The FACTS (Follow-on African Consortium for Tenofovir Studies) 001 study, a phase III trial that is testing the same regimen as was used in the CAPRISA 004 is on-going. There is a considerable expectation that soon an efficacious product will be available [17]. Modelling studies using data from South Africa suggest that even at low rates of use, the Tenofovir gel could be highly cost-effective, if it is proven to be an efficacious microbicide [18-20].

The acceptability of microbicide among the women in a given population is likely to be a major factor that will determine its effectiveness. In spite of far-reaching developments towards producing an efficacious microbicide, it is only in a limited number of settings that its likely acceptability has been evaluated. This could be an important drawback given that issues of human sexuality are often influenced by a complex interplay of disease, pregnancy risk perceptions, culture and gender norms regarding sexual practices and relationship dynamics [20,21]. Unless the acceptability of microbicide is evaluated concurrently with efforts at developing an efficacious product, its eventual deployment is likely to be fraught with challenges that could undermine its effectiveness [21,22].

With a national adult HIV prevalence of 1.5%, Ghana is among countries in the West African region with generally low HIV prevalence. There are however some sub-populations with considerably higher HIV prevalence. The prevalence of HIV among stationary sex workers, mobile sex workers and men who have sex with men have been found to be 52%, 37% and 25% respectively [23,24]. The patriarchal nature of traditional Ghanaian society also makes women in rural Ghana particularly vulnerable to HIV [24]. Preventing new infections is a major national priority. Ghana is among countries in sub-Saharan Africa that are likely to rapidly deploy an effective microbicide once it is approved internationally. Very little is known about the likely acceptability of microbicide among women in Ghana [23,25,26].

We conducted a study to assess microbicide acceptability among rural women in Ghana. Our objective is to contribute to the smooth incorporation of microbicides use in HIV prevention programs in the country once an efficacious product is approved.

## Methods

### Study setting

The study was undertaken in Kintampo, the municipal capital of the Kintampo North municipality of the Brong Ahafo Region of Ghana. This municipality lies at the exact geographical mid-point of Ghana and covers a land area of 5108 square kilometres. Most of the municipality is rural, with subsistence farming being the major occupation of the 96,358 inhabitants. A substantial proportion of the population are either migrant from the northern parts of the country or government workers who have been posted from the south. The strategic importance of Kintampo derives from the fact that it is a major stopover for passengers and haulage trucks plying the transnational highway between Ghana’s seaports in the south and its northern belts, including landlocked countries such as Burkina Faso, Mali and Niger. The Kintampo Lorry Park remains a beehive of commercial activity; day and night, seven days in the week. Anecdotal evidence suggests that commercial sex work thrives well in Kintampo.

### Study population

Study subjects were women who were aged between 18 and 40 years who were resident in either Kintampo or adjoining small towns and villages. The procedures for data collection consisted of both quantitative (questionnaire survey) and qualitative (focus group discussions) approaches.

### Quantitative approach

A questionnaire survey was conducted among pregnant women who were attending routine antenatal clinics in two health facilities in the Kintampo municipality over a 2-month period in 2010. The facilities are the Kintampo Municipal Hospital, which is a public health facility, and the Prince of Peace maternity home, which is a private health facility. All women aged 18–40 years were targeted to be interviewed. The questionnaire explored the socio-demographic background of respondents and their partners, their awareness and attitude towards the development of microbicides, their acceptability and perceived acceptability of their partners. Majority of the questions were closed-ended. The questionnaire was finalised after pre-testing at the same facilities in the week prior to the conduct of the actual study. They were administered verbally by research assistants who were trained to explain microbicides in the context of heterosexual contact, how it will be applied and the anticipated benefit of protecting against HIV infection. Research assistants explained microbicides as: “*Compounds that can be applied inside the vagina to protect against Sexually Transmitted Diseases (STDs) including HIV”.*

Data from the survey was double-entered, verified and cleaned using Microsoft Access. Analysis was done using STATA version 10. Analysis was performed to describe the socio-demographic background of respondents, proportion of respondents accepting to use microbicide and the preference for various formulations and other attributes. Univariate and multivariate analysis using logistic regression were performed to determine the factors associated with the acceptance of microbicide. Statistical significance was accepted when P-value was less than 0.05.

We targeted to interview 500 pregnant women and expected that this sample will afford the estimation of the level of acceptance of microbicides with a margin of error of 4% (at 95% confidence level), assuming that microbicides was acceptable to 50% of respondents.

### Qualitative approach

The preliminary findings of the survey were used to generate thematic areas that were explored further using focus group discussions (FGDs). Three FGDs were conducted with women aged 18–40 years living within the Kintampo municipality. Discussion groups were made of 8–12 people of about the same age. With age as a guiding criteria, women were classified into three categories; 18–25, 26–32, 33–40 years. The discussions were facilitated by a note taker and a moderator who used a guide developed from preliminary analysis of the quantitative survey, and related issues in the literature. All discussions were conducted in the local dialect and tape-recorded. They were then transcribed, translated from the local language into English and typed. The transcripts were read and responses grouped according to the thematic areas generated from the survey. Additional themes were added to the coding framework as determined by the data. The qualitative data was reviewed by two persons. To ensure inter-coder agreement and reliability 10% of the transcripts were coded independently and this was followed by a debriefing sessions to discuss the level of consensuses. Inter-coder reliability was always above 90%. A framework approach was adopted in the analysis which was performed using the software, QSR Nvivo (version 8).

### Ethical consideration

Ethical approval for the conduct of the study was obtained from the Institutional Ethics Committee of the Kintampo Health Research Centre. Administrative approval was also obtained from authorities of the two facilities where the study was conducted. Written Informed consent was sought from each respondent. Women were informed that they were free to decline to participate or withdraw. They were also assured of the confidentiality of the information they provide.

## Results

The findings of the survey and the focus group discussions were triangulated and are reported here with percentages that refer to findings from the survey and quotations that have been extracted from the FGDs.

A total of 504 women were surveyed. Their mean age was 26.0 years (stdev. 5.9). While 37% of women had had no formal education, 5.0% had had education beyond senior high school. Most of the women were Christians (71%) and petty traders (40%). Over half (59%) of them were married. About a third (34%) had no children, while 26% had only one child. The majority (55%) of them lived within the Kintampo Township. Only 11% of women lived outside of the Kintampo municipal. In univariate analysis, the acceptability of microbicide was found to be influenced by perceived partner acceptability (O.R. =11.5, 95% C.I. 3.65-36.24), perceived feasibility of discreet use (O.R. =9.77, 3.78-25.27), religion (O.R. =2.67, 1.16-6.12), age of partner (O.R. =0.31, 0.13-0.78) and whether the partner had children beside those with the woman interviewed (O.R. =0.42, 0.18-0.96) (Table 1). In multivariate analysis however only perceived partner acceptability (O.R. =17.7, 5.03-62.5) and perceived feasibility of discreet use (O.R. =8.9, 2.63-30.13) emerged as significant independent predictors of microbicide acceptability.

**Table 1:** Univariate analysis of the association between various factors and the acceptability of vaginal microbicides

**Characteristics**		**Acceptability**		**0R (95% C.I.)**	**P-value**
		**Yes**	**No**		
**Socio-demographic characteristics of respondents**					
Age (yrs)	>=26	202	14	0.52 (0.23-1.20)	0.12
	<26	278	10		
Highest educational level	Beyond primary	232	8	1.87 (0.78-4.47)	0.15
	Primary	248	16		
Ethnicity	Non-Akan	312	18	0.62 (0.24-1.60)	0.32
	Akan	168	6		
Religion	Christian	344	12	2.67 (1.16-6.12)	0.02
	Muslim	129	12		
Occupation	Skilled	61	1	4.56 (0.59-35.13)	0.11
	Non-skilled	254	19		
No of children	At least one	315	19	0.50 (0.18-1.37)	0.71
	None	165	5		
Place of residence	Outside of town	163	11	0.61 (0.26-1.45)	0.26
	Kintampo town	266	11		
**Socio-demographic characteristics of partner**					
Age (yrs)	>=34	207	17	0.31 (0.13-0.78)	0.01
	<34	272	7		
Highest educational level	Beyond primary	296	12	1.61 (0.71-3.66)	0.25
	Primary	184	12		
Ethnicity	Non-Akan	340	19	0.64 (0.23-1.75)	0.38
	Akan	140	5		
Religion	Christian	315	11	2.60 (1.11-6.08)	0.02
	Muslim	132	12		
Occupation	Skilled	105	4	1.63 (0.54-5.00)	0.38
	Non-skilled	289	18		
**Issues of marital relationship**					
Years of marriage	>=5	252	13	0.94 (0.41-2.13)	0.87
	<5	228	11		
Nature of marriage	Traditional rites	195	11	1.97 (0.78-4.96)	0.14
	Church rites	81	9		
Stay in same house	Yes	327	19	0.56 (0.21-1.54)	0.26
	No	153	5		
Has children with other women	Yes	109	10	0.42 (0.18-0.96)	0.03
	No	370	14		
Previously married	Yes	49	5	0.43 (0.15-1.22)	0.10
	No	429	19		
Has other wife(s)	Yes	54	3	1.13 (0.32-3.91)	0.85
	No	426	21		
**Likely attitude towards microbicides**					
Partner will find acceptable	Yes	351	5	11.51 (3.65-36.24)	<0.01
	No	61	10		
Ability to use secretly	Yes	389	7	9.77 (3.78-25.27)	<0.01
	No	91	16		

### Awareness

Only 2% of women were aware of the development of vaginal microbicide. In terms of familiarity with surrogates, 37% of women had had experience with the use of spermicides, 19% with lubricant use during sexual intercourse and 7% with other substances inserted into the vagina to enhance sexual pleasure.

### Comparison with other products

During the FGDs, many women likened the action of microbicide to how contraceptives and lubricants worked to prevent pregnancy and to enhance sexual pleasure respectively. Women who found it difficult to appreciate the mechanism of action of microbicides used contraceptive methods, sexual lubricants and fungicidal suppositories as reference materials. Such misconceptions were typically put as follows

“The way you have explained it, if a woman wants contraceptive protection, she should go for it. So yes, we can use it”

“.You know sometimes you as a woman is tired of child bearing, but the man wants more children. When this happened you will be able to hide and use it”

“Sometimes some people sell some drugs that people buy and insert for the men to enjoy sex with them”

“I know of a foaming tablet that can last for an hour. I don’t know if that is what you are talking about”

“I have used something like that before. It is like a seed. I had to insert it. It was for treating white (candidiasis)”

Only 1% of women indicated there were taboos in their cultures against the insertion of fingers into the vagina. Further enquiry during the FGDs did not yield new information on the existence of such taboos in the local culture. However a few women had difficulty reconciling the approach to using a microbicide with the health advice against douching. Some illustrative quotes are:

“What I know is that some nurses will tell you not to insert your fingers in the vagina; even washing the area is not advised since they say the vagina has a natural way of cleaning itself”

“I have not heard anything like that but it is more like putting drugs there that is not encouraged”

### Formulation preferences

Gel or creams were the most preferred (68% of women) microbicide formulation. In the FGDs however, concerns were expressed about the wetting effect of gels and creams, and how microbicides could affect sexual pleasure. For these reasons, some women wished that microbicides were tablets that could be swallowed. Such views were typically put as follows:

“I will like it in the form of a tablet to be swallowed because using a cream on a vagina will make the place wet, uncomfortable or some way”

“It should be a tablet to be swallowed because in the vagina it will melt and make the vagina soggy”

“..it is important that the vagina is tight. It makes you enjoy the sex better”

Compared to non-perfumed, red cream-coloured microbicide, fifty eight (58%) of respondents preferred perfumed and white microbicide.

### Acceptability

The overwhelming majority (95%) of women indicated they will be willing to use a microbicide if it became available. This extent of overwhelming acceptability was confirmed during the FGDs; typically put:

“Even if it becomes available this month we will really be happy”

Places where women felt they will be comfortable to receive free microbicides were health-related facilities (89-97% of women) such as chemical shops and hospitals, cosmetic shops (39%) and supermarkets (26%). The majority of women considered food vending spots as the least comfortable places to obtain free microbicides. For use in one round of sexual intercourse, the cost of a microbicide that will be considered affordable was US$0.75 by 49% of women and $1.50 by 15%. Majority (66%) of women would prefer to get microbicides from female vendors. Nearly all (99%) of the respondents preferred that such vendors be either of the same age (48%) or older (51%), and be persons they were not socially familiar with.

### Convert use

About 71% of women thought that their partners will find their use of microbicide acceptable, while 21% thought they would not be able to use it discreetly without their partners becoming aware. Apprehension about the feasibility and consequences of discreet use was apparent in the contribution of most women in the FGDs.

“For some men they will never agree for you to use it. If it were pills you could use it without his knowledge”

“If its pills you can use it secretly”

“No I cannot hide it from him”

Beside the effect of preventing HIV infection, 49% of women were concerned about the possible effect of microbicide on sexual pleasure. A quote by one woman in a focus group discussion illustrates this:

“Sexual pleasure is very important to men, you know some men have sex with some women and they say they enjoyed because her vagina is tight and the other way round. So prevention is important but sexual pleasure is paramount.”

## Discussion

Until recently the appropriateness of research on microbicide acceptability was questioned on the basis of the absence of an approved product [21,27,28]. However the need for such research among a wider population of potential end-users has now become urgent because of the significant progress that has been made in the development of an efficacious product. Such research is essential for generating useful information about potential user preferences including product characteristics such as formulation, colour, odour, feel, ease of use and impact on sexual pleasure. These attributes need to form an important part of microbicide development and the strategies for its promotion [21,23,29].

In the absence of a marketed product and away from a microbicide trial setting, the form of microbicide acceptability evaluated in our study could be described as perceived acceptability, i.e. satisfaction with the product and willingness to use it in the future or recommend it to others [21]. In this regard, the perception of respondents is based on their understanding of the product as explained by the investigators and on the basis of experience with microbicide-like products. It is conceivable that perceptions could change after experience with real products when they become available.

Our finding of a possible association between religious persuasion and microbicide acceptability coincides with the findings of *Hoel N et al. 2011*, who conducted a qualitative study to assess the reflections of Muslim women on the acceptability of vaginal microbicidal products in South Africa [30]. Although we failed to explore this further in the FGDs, we believe that it should prompt attention to the possibility that religious sensitivities could impact on the acceptability of microbicide among some sections of the society.

One of the important justifications that continue to drive interest in microbicides development is the consideration that it is a female-initiated product which can be used covertly. In many settings in sub-Saharan Africa, women are vulnerable to the possibility of partner violence, relationship termination, with attendant loss of financial support and social status [30]. As the findings of our study suggest covert use is likely to fester mistrust particularly when the male partner gets to find out. Limited education, limited employment opportunities and low income even if employed, force many women in sub-Saharan Africa to have men in their lives to ensure their survival [31]. Thus most women in this part of the world are not socially well-placed to take such “risk” [32]. Our finding that microbicide acceptability was strongly associated with perceived male partner acceptability is therefore not entirely unexpected. While many women may wish that it were possible to use a microbicide covertly, it does not appear that they consider it to be feasible. Among women who were reported to find microbicide acceptable in three sites in Africa and India, concerns about covert use were tied to issues of partner acceptability and preference, wetting effect, faithfulness and questionable health status [33]*.* Again, several studies have documented fear of abuse and other serious consequences especially in patriarchal societies where women generally have low assertive skills to negotiate safe sex [33-35]. The message of discreet use is therefore unlikely to be socially acceptable to women in formal and relatively stable relationships. It is unlikely to be a convincing enough message to rely upon in the promotion of microbicides. There is evidence to suggest that women would actually wish to be able to tell their partners and have their support [36-38]. In a study in Uganda that was linked to a microbicide trial, it was reported that whereas women during the pre-trial period believed that surreptitious microbicide use was advantageous, after the first week of product use, only 40% had actually been able to use the product secretly, 27% after 5 weeks, 22% after 10 weeks, and 13% after 5 months [38]. In other similar studies, even though women found microbicide gel highly acceptable, consistent use was tied to partner related factors which emphasises the need to adequately address partners acceptance in microbicide promotion [33,39].

In the promotion of microbicides, messages that promote its advantages for male partners need to be highlighted. An example is the reduced infectivity of HIV-infected women during heterosexual contact. For uninfected male partners, the use of microbicides should be promoted as being mutually beneficial in reducing, the risk of HIV infection for both male and female partners. Consistent microbicides should be promoted as the best available alternative for male partners who are reluctant to use condoms.

Sexual pleasure has emerged as an important consideration related to microbicide acceptability [40,41]. The values that societies place on sex vary across different cultural settings. In many cultures in sub-Saharan Africa, a norm has been established that females have the greater responsibility to satisfy the sexual pleasures of their partners [32,39]. This burden is considered greater when the woman finds herself in a polygamous marriage or when she has cause to believe that the male partner has other partners. It would appear unreasonable to ask a woman to use a product that could undermine her ability to satisfy her male partner sexually. In a study in Africa and India, acceptability of microbicide gel among women was linked to its lubricating effect and its consequent effect on sexual pleasure [33]. Several microbicide studies have reported how gender power affects the balance between the desire for sexual pleasure and acceptability of microbicide [34,39,42]. Our finding that nearly half of the women were concerned about the effect of microbicides on sexual pleasure is therefore not entirely unexpected. It corroborates similar reports from other studies, including those conducted outside sub-Saharan Africa [43]. In one of the failed microbicides trials, women were reluctant to return unused gel which had been given to them, even in the context of possible harm; because they reported that it made sex more pleasurable [29]. Any microbicide that adversely affects sexual pleasure is unlikely to be found acceptable, regardless of its efficacy. On the other hand, an efficacious product that has the added property of enhancing sexual pleasure is likely to be considered acceptable and more likely to be used consistently. Achieving a formulation that has the right biophysical and rheological properties to guarantee efficacy and sustain sexual pleasure should therefore remain a twin goal in microbicides development.

### Study limitations

A hypothetical microbicide was described to respondents in the study, most of whom were unfamiliar with the use of microbicides. In addition, some respondents may have considered some of the questions posed to be culturally sensitive. In spite of training to ensure uniformity, it is conceivable that respondents may have been influenced by the way the interviewers described the action of microbicides, and or how they posed the questions.

All the respondents in the study were women living in rural areas in Ghana. It is possible that women living in urban areas could have different perceptions of microbicides. The study was carried out in the wake of the release of the results of the CAPRISA 004 trial. The field of microbicide development is however evolving rapidly and as yet no microbicide has been approved by the World Health Organisation. Contradictory results have also emerged in other trials.

## Conclusion

The eventual availability of an approved microbicides is likely to be welcome news to rural women in Ghana. Belying such enthusiasm however are contextual issues such as religious persuasion, effect on sexual pleasure and perceived partner attitude. Unless such issues are addressed in product development and promotion, apparently high levels of microbicide acceptability are unlikely to be matched by consistent use.

## Competing interests

The author(s) declare that they have no competing interests.

## Authors’ contribution

MAA and FB were responsible for protocol development, study implementation, data analysis, drafting of manuscript and report-writing. SAA and KA were part of research team and contributed to study implementation and analysis of qualitative data. GA was responsible for quantitative data entry, data cleaning and analysis. SN, CT critically reviewed manuscript. All authors read and approved the final manuscript.

## Pre-publication history

The pre-publication history for this paper can be accessed here:

http://www.biomedcentral.com/1472-6874/12/40/prepub
